# Potential Cytotoxicity of Orthodontic Aligners in the Oral Environment: A Scoping Review

**DOI:** 10.3390/ma19091774

**Published:** 2026-04-27

**Authors:** Joanna Laskowska, Anna Paradowska-Stolarz, Marcin Mikulewicz

**Affiliations:** Division of Facial Abnormalities, Department of Maxillofacial Orthopaedics and Orthodontics, Faculty of Dentistry, Wroclaw Medical University, 50-425 Wroclaw, Poland; j.laskowska@umw.edu.pl (J.L.); marcin.mikulewicz@umw.edu.pl (M.M.)

**Keywords:** aligners, cytotoxicity, monomer release

## Abstract

Background: Clear aligner therapy (CAT) is increasingly popular due to aesthetic and functional advantages. Recent advances allow direct 3D printing of aligners, raising questions about their cytotoxicity and biocompatibility under intraoral conditions. Objectives: To review and synthesize current evidence on the cytotoxicity and biocompatibility of 3D-printed and thermoformable orthodontic aligners, and to identify factors affecting cellular responses. Eligibility criteria: Original research published from 2021 to 2026 evaluating the safety of orthodontic aligners; conference abstracts, editorials, and review papers were excluded. Sources of evidence: Medline (via PubMed), Scopus, Web of Science, and the Cochrane Library; search terms “aligner” AND “biocompatibility”; last search conducted on 31 January 2026. Charting methods: Data on authors, publication year, material type, experimental model, cytotoxicity assessment (extraction solvent, incubation period, cell line, and cell exposure), and main results were extracted independently by two reviewers. Results: Fourteen in vitro studies were included, seven on thermoformable aligners and seven on directly 3D-printed aligners; no clinical trials were identified. Material composition, post-processing, and oral environmental factors influenced cytotoxicity. Some materials exhibited acceptable biocompatibility, whereas others showed varying cytotoxic effects, indicating inconsistencies across studies. Conclusions: Directly 3D-printed and thermoformable aligners show potential for safe intraoral use, but evidence is limited to in vitro studies. Further standardized in vitro and in vivo research is needed to reliably assess cytotoxicity and ensure patient safety before widespread clinical implementation.

## 1. Introduction

Clear aligner therapy (CAT) originated from Kesling’s 1946 [1] concept of using sequential thermoplastic appliances to achieve gradual tooth movement, which later laid the foundation for modern clear orthodontic systems. The clinical introduction of clear aligners is generally dated to 1998, following FDA approval of the Invisalign system by Align Technology^®^. CAT offers several advantages over fixed appliances, including improved aesthetics, greater comfort, and easier oral hygiene [2,3]. Nevertheless, the biomechanical effectiveness of CAT remains highly dependent on the intimate fit of the aligner to the tooth surface. While CAT demonstrates predictable outcomes for movements such as alignment, leveling, anterior intrusion, and control of posterior buccolingual inclination, less predictable movements—including anterior extrusion, rotation of rounded teeth, torque expression, correction of anterior buccolingual inclination, and anchorage control—remain challenging. These limitations are largely attributed to insufficient force delivery resulting from suboptimal aligner–tooth contact. Consequently, this has driven the development of advanced three-dimensional (3D) printing technologies, as directly printed aligners (DPAs) exhibit improved surface adaptation, enhanced force transmission, and greater biomechanical efficiency compared to conventionally thermoformed aligners [4,5].

Beyond mechanical performance, CAT raises important biological and environmental concerns. Most aligners are fabricated from non-biodegradable thermoplastic polymers, contributing to plastic waste and microplastic pollution. Intraoral aging may lead to material degradation and the release of chemical substances as well as micro- and nanoplastic particles that can be ingested. Growing evidence links microplastic exposure to inflammatory, oxidative, immunological, and systemic effects, which is particularly relevant given the prolonged and often early-life exposure associated with CAT. Moreover, the single-use nature of aligners and their classification as medical waste add to their environmental burden [6,7]. In light of these considerations, evaluating the biological safety of aligner materials—especially their potential cytotoxic effects—has become increasingly important, forming the basis for the following review.

## 2. Methods

This study was conducted as a scoping review in accordance with the PRISMA-ScR guidelines [8]. The aim was to map the available evidence regarding the potential release of chemical substances from clear aligners in the oral cavity environment, identify the methodologies applied, and determine existing research gaps. A comprehensive electronic search with no language restrictions was performed in Medline (via PubMed), Scopus, Web of Science, and the Cochrane Library; eligibility was restricted to publications from 2021 to 2026. The search strategy was based on the keywords “aligner” AND “biocompatibility”. The most recent search was conducted on 31 January 2026. Eligibility criteria comprised original research, irrespective of study design, that evaluated the compliance of orthodontic aligners with requirements for safe intraoral use. Conference abstracts, editorials, and review papers were excluded. The following data were extracted: authors and year of publication, type of material, experimental model, cytotoxicity assessment method (including extraction solvent, incubation period, cell line, and cell exposure to potential toxic agents), and results. This review was registered on the https://osf.io/ch5u9 (accessed on 17 April 2026).

## 3. Results

**Figure 1:**
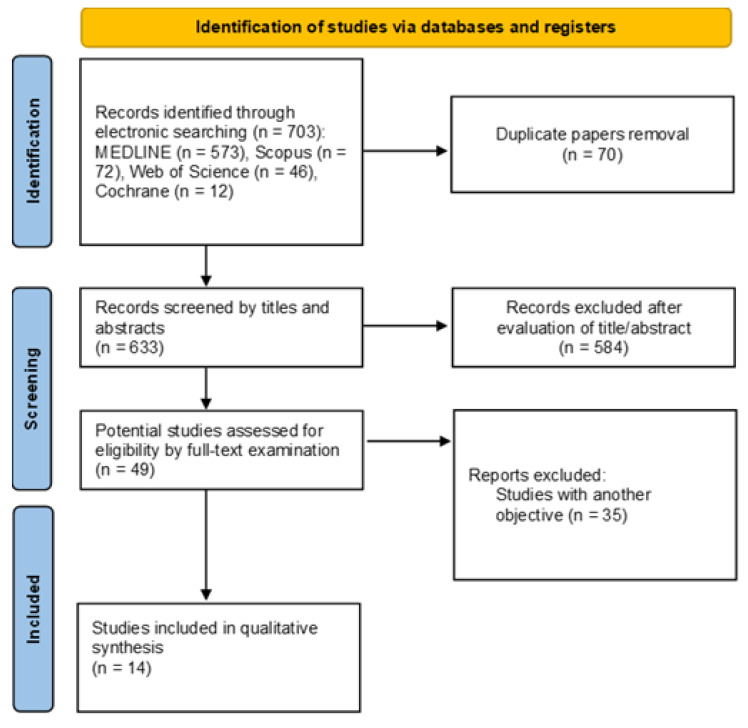
Flow diagram prepared in accordance with the PRISMA guidelines.

The workflow of the study screening and selection is depicted in Figure 1. After duplicates were discarded and the predefined exclusion criteria were applied, a total of 14 papers (listed in Supplementary Table S1) were deemed eligible and included in the review.

### 3.1. Study Characteristics

Among the research covered, seven [9,10,11,12,13,14,15] examined thermoformable aligner materials, whereas seven [16,17,18,19,20,21,22] explored directly printed aligners. All fourteen investigations employed in vitro experimental designs, and no clinical trials were identified.

#### 3.1.1. Cytotoxicity of Thermoformed Aligners

Among the studies investigating thermoformed aligners, a range of commercially available systems and materials were evaluated. These included:

- Invisalign^®^ (Align Technology Inc., San Jose, CA, USA) [9,14,15];
- Eon Clear Aligners^®^ (Eon Holdings, Amman, Jordan) [9];
- Clarity^TM^ (3M Oral Care Solutions, St. Paul, MN, USA) [9];
- Sure Smile^®^ (Dentsply Sirona, Richardson, TX, USA) [9,14,15];
- Angel Aligner^TM^ (Angelalign Technology Inc., Shanghai, China) [10];
- Zendura^TM^ (Bay Materials LLC, Fremont, CA, USA) [11,13];
- Clear Correct^®^ (Straumann Holding AG, Basel, Switzerland) [11,15];
- Duran^®^ (Scheu-Dental GmbH, Iserlohn, Germany) [13];
- Essix^®^ (Dentsply Sirona^®^, Fair Lawn, NJ, USA) [13];
- Leone^®^ (Leone S.p.a. Orthodontic and implantology, Firenze, Italy) [13];
- Maxflex^®^ (Cuumed Catheter Medical, Taichung, Taiwan) [13];
- Keystone^®^ (Keystone^®^ Industry, Gibbstown, NJ, USA) [13];
- Spark^TM^ (Ormco Corporation, Brea, CA, USA) [14].

In the study by Yu et al. [12], the manufacturer was not specified; however, the material was identified as thermoplastic polyurethane (TPU). Additionally, in a study by Yan et al. [10], a fluoride-coated variant of Angel Aligner was evaluated alongside the conventional material.

While the majority of studies focused exclusively on thermoplastic materials, Bethala et al. [14] conducted a comparative analysis of the cytotoxicity of thermoplastic materials—Invisalign, SureSmile, and ClearCorrect—against that of 3D-printed aligners fabricated from TC-85 DAC^®^ (Graphy, Seoul, Republic of Korea).

Two studies assessed non-thermoformed materials [10,12], while three investigations examined finished aligners [9,11,14]. Conversely, the research conducted by Lo et al. [13] and Bethala et al. [15] investigated both original thermoplastic aligner sheets and thermoformed samples.

The included studies had varying sample sizes. In research on non-thermoformed materials, sample sizes were reported as follows: rectangular specimens of 2.4 cm × 2.4 cm [10], disk-shaped specimens with a diameter of 3.5 mm (3.2 g, 0.1 g/mL) [13], and circular disks with a diameter of 4.5 mm [15]. In the study by Yu et al. [12], the specimen size was not mentioned; instead, the samples were characterized by mass, with an estimated weight of 6 mg. In studies evaluating finished aligners, variability in specimen size was also observed. In study [13], the aligner material was punched from thermoformed sheets into disks with a diameter of 3.5 mm, with a total material mass of 3.2 g used. In study [14], square specimens measuring 10 × 10 × 10 mm were prepared from the aligners. In contrast, study [15] used round transparent specimens with a diameter of 4.5 mm. The studies by Alhendi et al. [9] and Dinu et al. [11] did not report the dimensions of the prepared specimens.

Only the research conducted by Lo et al. [13] delineated a disinfection protocol for the aligners following their manufacturing and preceding incubation. The authors indicated that the aligners were submerged in 75% alcohol; however, the time of the immersion was not detailed.

The cytotoxicity of the thermoplastic materials was evaluated utilizing cell cultures. Six studies [9,10,11,12,13,15] employed an indirect contact assay, evaluating the effects of eluates derived from thermoplastic materials on cell cultures using the following media:

- Dulbecco’s Modified Eagle’s Medium (DMEM) [12,13,15];
- Alpha MEM [10];
- Normal saline solution [9];
- Artificial saliva [11].

The amount of media was specified as a 0.1 g/mL ratio in studies [13,15], and as 3 cm^2^/mL in study [12]. All samples were incubated at 37 °C. The duration of eluate incubation prior to analysis varied among studies: 1 month [9], 3 days [10], 7 days [11], and 24 h [12,13,15].

One study [14] assessed monomer release directly into the culture medium using a direct contact assay, in which cells were cultured directly on the surface of the samples.

Cytotoxicity was assessed in nearly all studies using undiluted eluates. Only Alhendi et al. [9] and Dinu et al. [11] formulated diluted eluates in culture medium or water prior to testing. The dilution ratios utilized were 5%, 10%, and 20% *v*/*v* [9], as well as 1:6, 1:4, 1:2, and 1:1 (sample to culture medium or water) [11]. The studies employed a variety of cell lines to assess cytotoxicity, including: human gingival fibroblasts (HGFs) [9,10,11], human epidermal keratinocytes (HaCaT) [11,12], mouse embryonic fibroblasts (NIH/3T3) [12], human periodontal ligament cells (HPDL) [13], human foreskin fibroblasts (HFF-1) [14], and mouse fibroblasts L929 [15].

In the studies conducted by Dinu et al. [11] and Yu et al. [12], two cell lines were used. Dinu et al. employed human epidermal keratinocytes (HaCaT) and mouse embryonic fibroblasts (NIH/3T3), whereas Yu et al. incorporated human gingival fibroblasts (HGFs). The effects of the eluates on cells were evaluated at multiple time points, including after 24 h [10,11,12,13,15], as well as on day 3 [12,13,14], day 5 [13], and on days 7 and 14 [15]. Cytotoxicity was assessed using viability assays such as MTT [11,13,15], CCK-8 [10,12], and resazurin-based tests [14].

Six [10,11,12,13,14,15] out of seven studies demonstrated no cytotoxic effect, with cell viability remaining above 70%. In contrast, the study by Alhendi et al. [9] reported moderate cytotoxicity, with cell viability ranging from 30% to 59% for Invisalign and SureSmile at 20% concentration, and for Eon at 10% concentration.

#### 3.1.2. Cytotoxicity of Directly Printed Aligners

Three commercially available resins have been evaluated for their biocompatibility in research on 3D-printed orthodontic aligners: Tera Harz TC-85 DAC^®^ (TC-85) [16,17,18,19,20,22], Tera Harz TA-28^®^ (TA-28) [21], and Clear-A^®^ (C-A) [22]. Furthermore, Iodice et al. [19] conducted a comparison of 3D-printed aligners fabricated from TC-85 resin against thermoformable aligners, utilizing Zendura FLX as the control material. Among studies evaluating the biocompatibility and cytotoxicity of directly printed aligners, one utilized an SLA-based system, the Ackuretta SOL [22]; the others employed DLP printers, including the Sprintray Pro 55^®^ (Sprintray, Los Angeles, CA, USA) [16,17], AccuFab-L4D^®^ (Shining 3D Tech., Hangzhou, Zhejiang, China) [18], Uniz Nbee^®^ (Uniz Technology, San Diego, CA, USA) [20], and the Asiga MAX 3D printer (Asiga SPS™ technology, Sydney, Australia) [21].

In certain experiments, specimens were constructed in standardized geometries, such as squares [19], disks [20], or rectangles [22]. Other studies directly assessed 3D-printed aligners in their original configuration by sectioning them into smaller specimens [17,18,20]. Campobasso et al. [18] reported specific printing parameters, including a thickness of 0.75 mm, a thickness of 0.5 mm, a printing angle of 45°, and a layer height of 100 μm.

Various post-curing techniques, according to the unique resin type, have been implemented across the included investigations to assess cytotoxicity. Clear-A resin was polymerised via the Curie Cure device (Ackuretta, Taipei, Taiwan) [22]. Different curing approaches for TC-85 DAC^®^ resin have been documented, including the application of the Cure M^®^ unit (Graphy, Seoul, Republic of Korea) for 24 min to each side [17] and the THC2^®^ device (Graphy) for durations of either 20 min [19] or 25 min [22]. Campobasso et al. [18] compared polymerisation results utilizing the THC2^®^ device for 14 min per side to those achieved with the FormCure^®^ device (Formlabs, Somerville, MA, USA) for 30 min per side. Iodice et al. [19] examined the impact of different curing lengths, namely 14, 24, and 50 min. Moreover, certain studies included additional post-curing treatments, such as ultrasonic cleaning in an 80 °C bath for 2 min, succeeded by 1 min in boiling water and subsequent drying [18], or ultrasonic cleaning in warm water for 1 min, followed by immersion in boiling water (100 °C) for an extra minute [22].

In all examined investigations, isopropyl alcohol (IPA) was used as the principal cleaning agent post-printing. Reported IPA rinse durations varied: 4 min in certain studies [17,19], 5 min in others [22], and 6 min in one study [18]. Bor et al. [22] initially cleansed the specimens with isopropyl alcohol and subsequently rinsed them with sterile deionised water. Furthermore, Kim et al. [20] assessed various cleaning techniques, encompassing ultrasonic washing in isopropyl alcohol for one minute, alongside centrifugation at ambient temperature and at 55 °C for durations of 2, 4, and 6 min.

The cytotoxicity of 3D-printed resin materials was evaluated utilizing cell cultures. Six studies [16,18,19,20,21,22] employed an indirect contact assay, assessing the effects of eluates derived from the printed resins on cell cultures using the following media:

- Dulbecco’s Modified Eagle’s Medium (DMEM) [16,18,19,21,22];
- RPMI 1640 supplemented with 1% antibiotic/antimycotic and 10% fetal bovine serum (FBS) [20].

All samples were incubated under standard cell culture conditions. The duration of eluate incubation prior to analysis varied among studies: 14 days [16,18,19], 12 days [21], 7 days [18], 72 h [22] and 24 h [20].

Cytotoxicity was assessed using a range of assays. The majority of studies employed viability and metabolic activity tests, including MTT [16,17,18,19,20], XTT [22] and resazurin-based tests [21]. The release of residual monomers from 3D-printed resins was examined utilizing sophisticated analytical methods. Specifically, Willi et al. [17] employed liquid chromatography tandem mass spectrometry (LC-MS/MS), facilitating the highly sensitive and specific identification of UDMA and BPA. Distilled water was used as the extraction medium, with an incubation period of one week prior to analysis.

The studies utilized various cell lines to assess cytotoxicity, including: mouse fibroblasts L929 [20], mouse fibroblasts 3T3 [18], and human gingival fibroblasts HGF-1 [16,19,21,22].

Three out of seven studies demonstrated slight cytotoxic effects of 3D-printed aligner materials. Cytotoxicity was influenced by the type of post-curing unit [17] and increased with longer curing times [19]. In contrast, four studies [16,20,21,22] reported no cytotoxic effects, indicating no significant influence of factors such as isopropyl alcohol (IPA) rinsing time, type of rinsing solution, or centrifugation during post-processing.

Additional methods included nuclear staining with Hoechst dye [19] and intracellular reactive oxygen species (ROS) measurement using the DCFH-DA method [16].

## 4. Discussion

Biocompatibility is a fundamental requirement in biomedical applications, as living tissues come into contact with a wide range of materials used in the production of medical devices, with exposure times dependent on their specific clinical roles. It is commonly defined as the ability of a material to fulfill its intended purpose within a biological environment without inducing detrimental effects on physiological functions or disrupting homeostasis. Traditionally, the evaluation of biocompatibility has focused largely on parameters such as cell viability and toxicity [23].

Some inorganic biomaterials used in medicine have been described as non-toxic, chemically inert, and suitable for biomedical applications due to their favorable physicochemical properties [24]. Potential cytotoxic effects observed in clear aligner materials result from a complex, multifactorial set of influences encompassing the chemical makeup of the polymers, subsequent material degradation, and external processing and environmental factors such as thermoforming and intraoral exposure. Central pathways contributing to this toxicity include the release of residual monomers or additives from the polymer matrix, degradation of the material surface, the generation of oxidative stress, and the activation of inflammation-related cellular responses [22,25]. The principal mechanisms reported in the literature specifically involve the leaching of unreacted monomers or formulation additives, the emission of degradation by-products from the material’s surface, and the stimulation of inflammatory responses directly attributable to interaction with the material [26].

Within the studies included in this review, only one was designed to analyze monomer release, employing liquid chromatography techniques: applied liquid chromatography coupled with tandem mass spectrometry (LC-MS/MS). A study conducted by Willi et al. detected UDMA at levels above the limit of quantification (LOQ), with the lowest concentrations observed in samples subjected to autoclaving at 132 °C [17]. This approach is conceptually aligned with earlier studies investigating the aging of thermoformed aligners [27], in which solvent extracts were analyzed using gas chromatography–mass spectrometry equipped with an electron-impact ionization detector. Those analyses revealed no detectable residual monomers or oxidative by-products. In those earlier experiments, aligners were immersed in a 75% ethanol/25% water solution for 2 weeks at 23 °C to simulate material aging.

According to EN ISO 10993-5 [28], biocompatibility testing is designed to ensure the safe and appropriate use of medical devices by identifying potential biological risks associated with dental prosthetic and orthodontic materials. These assessments are conducted using in vitro or non-animal in vivo approaches to detect adverse biological responses that may result from prolonged interaction between the device and human tissues, with the evaluation of acute, subchronic, and chronic toxic effects—potentially caused by residual manufacturing substances, post-processing procedures, or external contaminants—carried out through a series of standardized tests, including cytotoxicity, genotoxicity, hemocompatibility, epicutaneous testing, as well as endotoxin and pyrogen assays. Within this framework, EN ISO 10993-5 specifically addresses in vitro cytotoxicity testing as a core element of biocompatibility evaluation, establishing criteria for assessing whether a material inhibits cell growth or viability when in direct or indirect contact with cultured mammalian cells, thereby informing the selection and risk mitigation of materials used in medical devices such as orthodontic appliances. In the context of contemporary dental materials research, an increasing body of in vitro evidence has examined the cytocompatibility and leachable compound profiles of clear aligners and related orthodontic devices, often aligning experimental extraction methodologies with ISO 10993-5 guidance and reporting generally favorable biocompatibility outcomes when protocols adhere to these international standards.

Polyethylene terephthalate (PET) is a polymeric ester compound formed by the dehydration and condensation of terephthalate (TPA) and ethylene glycol (EG; CAS RN 107-21-1). The chemical structure of PET consists of TPA and EG units connected by ester bonds, along with aromatic ring structures. The production method and supplier of PETG significantly impact the cytotoxicity and biocompatibility of the material. For example, in comparison to Biolon brand PETG, Duran PETG had a 20% difference in cell viability, with an 84.6 ± 4% viability [29]. This phenomenon is consistent with all medical implants and devices, given that the manufacturing of the product can greatly affect the biocompatibility, and the material should be sourced accordingly.

In the research conducted by Bethala et al. [15], ClearCorrect exhibited the highest cell viability, followed by SureSmile, Graphy, and Invisalign on both Day 1 and Day 7; nonetheless, these variations lacked statistical significance.

In the study conducted by Lo et al. [13], the effect of thermoforming on cell viability varied: for PETG (Duran, Essix, Leone), TPU (Zendura, Maxflex), and PET (Keystone), certain samples immersed for 14 days showed slightly reduced viability in the thermoformed specimens, which was statistically significant.

Aligners are manufactured through direct 3D printing using several resin types. The resins utilized in direct aligner production often consist of a blend of photoinitiators, additives, and monomers, including acrylates and methacrylates (e.g., UDMA, HEMA). These components resemble those present in the chemical formulation of acrylic resins utilized for removable orthodontic devices, as well as in adhesives and sealants for fixed orthodontic appliances. Initially, the study concentrated on resins such as Dental LT^®^ (Formlabs, Somerville, MA, USA) [30,31,32], E-Guard^®^ (EnvisionTEC, Dearborn, MI, USA) [32], and Freeprint^®^ Splint 2.0 (Detax, Ettlingen, Baden-Württemberg, Germany) [23], which were designed for stiff appliances like splints and retainers rather than for aligner fabrication. The resins evaluated in those experiments exhibited favorable biocompatibility, with no harmful effects detected. Furthermore, Fayyaz Ahamed et al. [31] evaluated the cytotoxicity of aligners manufactured with Dental LT^®^ resin (Formlabs, Somerville, MA, USA), affirming similar biocompatibility profiles to thermoformable aligners. Recently, focus has transitioned to the TC-85 photo-polymerizable polyurethane resin, indicating its increasing prevalence in 3D-printed aligner applications. DPAs composed of Graphy’s resin TC-85 have attained CE and KFDA certification, in addition to FDA approval [32].

3D-printed aligners are produced using 3D printers that utilize VAT photopolymerization technology (SLA), encompassing SLA, DLP, and LCD systems—the DLP variant being the most common. Each printer comes with dedicated software for arranging models and adding supports, which plays a key role in achieving precise prints. Models can be printed horizontally (enabling faster printing but requiring more supports and allowing fewer models per run) or vertically (taking longer but using fewer supports and fitting more models per run, at the risk of more printing errors). The printing process is typically conducted at a z-axis resolution of 100 μm. To ensure reliable results, the resin must be properly agitated and maintained at around 30 °C to avoid print defects [33].

Previous studies have demonstrated that post-processing conditions—such as choice of cleaning agent, washing duration, and curing procedure—significantly influence both the mechanical performance and the cytotoxicity of 3D-printed dental resins [25]. These findings lay an important foundation for understanding the cytotoxic potential of directly 3D-printed aligners made from similar photopolymer materials. Because as-printed aligners do not immediately exhibit optimal mechanical properties or biocompatibility, a series of post-processing procedures is required, analogous to the finishing steps in conventional aligner manufacturing. Post-processing protocols are designed to promote complete resin polymerization, thereby minimizing residual monomers (the degree of conversion in resin-based materials rarely reaches 100%—typically ~75% in most composites [34] and ~83% in TC-85 DAC resin [17]). These protocols also aim to ensure that the material’s structure can resist degradation during aging in the oral environment. The first stage typically involves the removal of any residual uncured resin from the printed surface. Subsequently, the support structures are detached either manually or with the use of nippers. The printed trays are then subjected to ultraviolet light curing. Research on dental resins has shown that the polymerization process depends on multiple factors. Importantly, with respect to biocompatibility, higher curing temperatures and longer curing times have been linked to increased cell survival and consequently lower cytotoxicity [35,36]. Sobti et al. found that extending the post-curing time reduces the amount of water absorbed by directly 3D-printed aligners [37]. After polymerization, a layer of unreacted monomer typically remains on the surface of 3D-printed components. The presence of this layer can lead to adverse biological effects, including inflammatory reactions upon contact with tissues, mitochondrial damage, and the proliferation of microorganisms responsible for dental plaque formation [38]. To mitigate these effects, the removal of this surface monomer layer is recommended.

The structural changes observed in aligners after actual intraoral use result from a far more complex environment. Saliva contains esterases capable of hydrolyzing the ester bonds within the resin matrix, leading to the release of monomers. This leaching process may be further intensified by intraoral heat, moisture, and masticatory forces, collectively accelerating material degradation. To more accurately assess monomer release and cytotoxicity under conditions resembling the oral environment, researchers have sought to replicate key intraoral factors, such as temperature and humidity, in vitro. A noteworthy study by Dinu et al. [11] explored the effect of saliva pH on aligner cytotoxicity by incubating aligner samples in artificial saliva at three pH levels: acidic (pH 3.3), neutral (pH 7.41), and basic (pH 9.0), using an orbital shaker to simulate movement. The authors reported a slight reduction in keratinocyte confluence under acidic conditions, suggesting that lower pH levels may enhance material toxicity in the oral environment. Nevertheless, cell viability and overall cell count remained above 70% across all pH conditions. Furthermore, reduced solution concentrations exhibited enhanced cell viability and significantly decreased cytotoxicity [9].

The aligner replacement interval, typically ranging from 7 to 14 days, is designed to balance efficient tooth movement with adequate adaptation of the surrounding periodontal tissues. A 7-day schedule may shorten overall treatment time and enhance patient comfort, whereas a 14-day interval can be advantageous in cases requiring more controlled movement or greater periodontal support. Evidence suggests that extending the change interval to two weeks increases the likelihood of successfully completing the initial aligner series compared with weekly replacement [39]. It is important to note, however, that aligners are typically replaced every 7–14 days, meaning that the potential source of cytotoxicity is repeatedly reintroduced into the patient’s oral environment.

## 5. Conclusions

Within this scoping review, the available evidence on the cytotoxic potential of thermoplastic and 3D-printed aligners is mapped with regard to their biocompatibility under intraoral conditions. The review emphasizes that a realistic simulation of the oral environment is essential for reliable evaluation. In the case of DPAs, post-processing techniques appear promising in reducing biological risks; however, further in vitro and in vivo investigations are required to substantiate current findings and to support the development of safer materials.

## Data Availability

No new data were created or analyzed in this study. Data sharing is not applicable to this article.

## Supplementary Materials

The following supporting information can be downloaded at https://www.mdpi.com/article/10.3390/ma19091774/s1, Table S1: Papers were deemed eligible and included in the re-view. Table S2. Scoping Reviews (PRISMA-ScR) Checklist.
