# Supplementary material for: Potential Cytotoxicity of Orthodontic Aligners in the Oral Environment: A Scoping Review
Source: Materials (Basel). 2026 Apr 27;19(9):1774. doi: 10.3390/ma19091774 (PMC13164491; doi:10.3390/ma19091774)
Supplement: Supplementary file 1 [file materials-19-01774-s001.zip › materials-4119619-supplementary.pdf]

Review

Potential Cytotoxicity of Orthodontic Aligners in the Oral Environment: A Scoping Review

Joanna Laskowska, Anna Paradowska-Stolarz * and Marcin Mikulewicz

Division of Facial Abnormalities, Department of Maxillofacial Orthopaedics and Orthodontics, Faculty of Dentistry, Wroclaw Medical University, 50-425 Wroclaw, Poland; j.laskowska@umw.edu.pl (J.L.); marcin.mikulewicz@umw.edu.pl (M.M.)

* Correspondence: anna.paradowska-stolarz@umw.edu.pl; Tel.: +48-(71)-7840299

Table S1. Papers were deemed eligible and included in the re-view.

Author/Year	Type of Tested Material	Study Design	Experimental Model	Cytotoxicity Assessment Method	Results
Alhendi et al. (2022)	Three sets of clear aligners (maxillary and mandibular trays) obtained from four manufacturers: Invisalign, Eon, Clarity, and SureSmile	In vitro	Each set of appliances was immersed in normal saline solution in glass containers for 1 month at 37°C. Eluent samples were subsequently diluted to 5%, 10%, and 20% (v/v), with 20% as the maximum concentration to avoid culture medium dilution.	Cytotoxicity was evaluated in human gingival fibroblasts (HGFs) cells) exposed to aligner extracts (5%, 10%, and 20%) for 48 hours. Cell viability was determined using the MTT assay by spectrophotometric measurement, and cytotoxicity was classified based on viability percentages.	The tested thermoplastic aligners (Invisalign, Eon, Clarity, and SureSmile) exhibited slight to moderate cytotoxicity in a concentration-dependent manner. Higher solution concentrations reduced cell viability and increased cytotoxicity, except for the Eon system, which showed a non-linear pattern. Clarity consistently maintained higher cell viability across all concentrations, while Invisalign and SureSmile showed slight cytotoxicity at 5% and 10% and moderate cytotoxicity at 20%. Eon demonstrated slight cytotoxicity at 5% and 20% and moderate cytotoxicity at 10%. Overall, no significant differences were observed among the four systems, and lower solution concentrations resulted in higher viability and lower cytotoxicity.
Yan et al. (2023)	FCAP (fluoride-coated) and CAP (standard) from Angelalign Technology Inc, China	In vitro	The materials were soaked in supplemented DMEM for 24 h at 37°C to prepare extracts, which were then	Cytotoxicity was evaluated using the CCK-8 colorimetric assay on human gingival fibroblasts (HGFs). After 24 h exposure to aligner extracts, CCK-8 reagent	The CCK-8 assay showed no significant differences in cell viability between CAP, FCAP, and the negative control group ($P > 0.05$). Live/dead staining confirmed high cell viability, with almost no dead cells observed in any group.

			applied to cultured cells for cytotoxicity assessment	was added and incubated for 2 h. Absorbance was measured at 450 nm using a microplate reader, and cell viability was calculated relative to the negative control group. Cytotoxicity was determined based on the reduction in metabolic activity compared with the control group.	The coating did not compromise the biocompatibility of the clear aligner.
Dinu et al. (2024)	Two thermoformed clear aligners: CA1 (Zendura, Straumann Group, Switzerland) and CA2 (ClearCorrect, Straumann Group, Switzerland).		Aligners were incubated in artificial saliva at three different pH values (acidic, neutral, and basic) for 7 days at 37°C in an incubator with an orbital shaker. After incubation, the appliances were removed and the saliva extracts were diluted in culture medium or water at ratios of 1:6, 1:4, 1:2, and 1:1	Cytotoxicity was evaluated in HaCaT (immortalised human keratinocytes) and HGF (human gingival fibroblasts) cell lines cultured under standard conditions. Cells were exposed to the tested samples at dilutions of 1:6, 1:4, 1:2, and 1:1, and viability was assessed after 24 hours using the MTT assay. Cell number was additionally determined by automated microscopy and image analysis.	Both tested clear aligners showed a dilution- and cell-dependent cytotoxic effect after 24 hours of exposure. Zendura slightly reduced the viability and number of HaCaT and HGF cells, with significant effects mainly at the 1:1 dilution, while gingival fibroblasts showed higher sensitivity than keratinocytes. ClearCorrect exhibited a slightly higher cytotoxic effect, significantly decreasing cell viability and number at lower dilutions, particularly in HGF cells. However, cell viability remained above 70% in all conditions, and the pH of the incubation media did not significantly influence cytotoxicity.
Yu et al. (2024)	TPU film	In vitro	The material was incubated in Dulbecco's modified Eagle medium (Gibco, USA) with 10% fetal bovine serum, 100 U/ml penicillin, and 100 μg/ml streptomycin (Gibco) for 24 hours at 37°C (3 cm^2/ml)	Cytotoxicity of the extract was tested with CCK-8 using NIH/3T3 and HaCaT cells	CCK-8 assay confirmed good biocompatibility of the TPU film, with no significant cytotoxicity differences between the extract and the negative control for both cell types. Hemolysis rate was ~1.04%, below the 5% threshold. Fluorescence images of NIH/3T3 cells after 3 days showed normal morphology in both culture medium and extract.
Lo et al. (2024)	Original and thermoformed thermoplastic aligner sheets including PETG (DURAN®, ESSIX®, Leone®), TPU	In vitro	Extract-based model using 3.5 mm discs punched from original and thermoformed aligner sheets (0.1 g/mL), disinfected in 75% alcohol and incubated in	Cell viability of HPDL cells was evaluated using MTT assay after 24 h exposure to material extracts; absorbance was measured at 540 nm and viability (%) was calculated relative to untreated	HPDL cell viability remained above 70% for all materials and conditions. Thermoforming influenced viability variably: for PETG (Duran, Essix, Leone), TPU (Zendura, Maxflex), and PET (Keystone), some 14-day immersed groups showed slightly lower viability in thermoformed samples ($P < 0.05$), while 7-

	(Maxflex™, Zendura®), and PET (Keystone®).		supplemented DMEM for 7 and 14 days prior to cell exposure.	controls. Morphology was additionally assessed by light microscopy.	day groups generally exceeded 70%. Cells maintained spindle-shaped morphology across all groups and time points (days 1, 3, 5) under 100x–400x magnification.
Martínez Gil-Ortega et al. (2025)	Commercial clear aligners: Spark (polyurethane resin containing 2-hydroxyethyl methacrylate and poly(oxy-1,2-ethanediyl) derivatives; 0.75 mm; Ormco Corporation, USA), Invisalign LD30 (multilayer aromatic thermoplastic polyurethane based on methylene diphenyl diisocyanate and 1,6-hexanediol; 0.75 mm; Align Technology Inc., USA), and SureSmile Smile Plus/C Plus (polypropylene/ethylene copolymer >95% with stabilizers <5%; 0.035–0.040 inches ≈0.9–1.0 mm; Dentsply Sirona, USA)	In vitro	Culture of human foreskin fibroblasts (HFF-1, ATCC) seeded (5×10^4 cells/sample) onto 10 × 10 mm aligner specimens (n=10 per aligner; triplicate conditions) and incubated for 3 days at 37 °C in DMEM supplemented with 15% FBS and 1% penicillin–streptomycin.	Resazurin-based metabolic assay after 3 days of incubation; fluorescence measured using a spectrophotometer (excitation 560 nm, emission 590 nm).	All tested aligners demonstrated cytocompatibility above 70%. ANOVA revealed statistically significant differences in cytotoxicity (p = 0.0037). The control group showed higher cell viability compared with Spark (mean difference = 1.351) and Smile (mean difference = 1.525), while no significant differences were observed among the clear aligners themselves.
Bethala et al. (2026)	Thermoplastic sheets of four clear aligners: Invisalign (Align Technology Inc, USA), SureSmile (Dentsply Sirona, USA), Clear Correct (Straumann AG, Switzerland), and	In vitro	Round discs (4.5 mm diameter, 0.75–1 mm thickness) from Invisalign, SureSmile, Clear Correct, and Graphy Aligners were incubated in DMEM at 37°C for 14 days at a 0.1 g/mL weight-to-volume	MTT assay using L929 fibroblast cells. Cells were exposed to extracts of commercial clear aligners for 24 h, then incubated with MTT dye (10 µL of 10 mg/mL) for 4 h at 37°C. Formazan crystals were dissolved in DMSO and absorbance measured at 570	No cytotoxicity was observed at Day 1, 7, or 14 (all groups: Nil). Across all time points, the highest cell viability was consistently recorded for Clear Correct, while the lowest was observed for Invisalign. Intergroup differences were not statistically significant (Day 1: p=0.445; Day 7: p=0.284; Day 14: p=0.055), except for Invisalign vs Clear Correct at Day 14 (p=0.044). Cell viability increased over time in all

	Graphy Aligners (Graphy, Korea).		ratio. Extracts were filtered to remove solids and stored at −20°C for further cytotoxicity and biochemical testing.	nm. Cell viability (%) was calculated relative to control.	groups (p>0.05). No fibroblast lysis was detected (L929, 20×).
Pratsinis et al. (2022) [24]	3D-printed clear aligners made of photo-polymerizable polyurethane resin (Tera Harz TC85A- Graphy, Seoul, South Korea)	In vitro	Aligners were sectioned and fragments pooled per patient, then immersed in 25 mL sterile deionized water and incubated for 14 days at 37°C. Deionized water without aligners served as negative controls. After incubation, eluates were collected, stored at −80°C, and adjusted to 0.9% NaCl prior to cell culture experiments.	Cytotoxicity was evaluated using a human gingival fibroblast cell line derived from a healthy donor. Cells were seeded in 96-well plates (25,000 cells/cm²) and, after overnight incubation, exposed to aligner eluates at final concentrations of 20%, 10%, and 5% (v/v) for 72 hours. Cell viability was determined using a modified MTT assay.	Eluates from 3D-printed aligners were not cytotoxic to human gingival fibroblasts at any tested concentration (20%, 10%, 5% v/v; P > 0.05). No significant effects on intracellular ROS levels were observed compared with negative controls (P = 0.08). Additionally, at 20% v/v, eluates did not induce proliferation of estrogen-sensitive MCF-7 cells (P = 0.65) and showed no difference compared with MDA-MB-231 cells (P = 0.78), indicating absence of estrogenic activity. Overall, no cytotoxic, oxidative, or estrogenic effects were detected.
Willi et al. (2023) [27]	3D-printed aligners made of photopolymerized Tera Harz TC85A resin	In vitro	Ten aligners were individually immersed in 10 mL of double-distilled water and incubated at 37°C for 7 days. Three water samples without aligners served as controls.	Eluates were collected and analyzed for urethane dimethacrylate (UDMA) and bisphenol A (BPA) content using liquid chromatography–tandem mass spectrometry (LC–MS/MS).	ATR-FTIR analysis identified the resin as an aromatic-free UDMA derivative, with a mean degree of conversion (DC) of 83 ± 3.6%. LC–MS/MS analysis confirmed the release of UDMA from all aligner specimens, with a mean concentration of 50.9 ± 15.6 μg/L. The method showed good linearity and sensitivity (LOD: 0.25 μg/L; LOQ: 0.75 μg/L). Bisphenol A (BPA) was not detected in any aligner eluates or control samples.
Campobasso et al. (2023) [32]	3D-printed orthodontic aligners (Tera Harz TC-85 DAC resin)	In vitro	DMEM with cultured cells were incubated with aligner specimens. Aligners were post-cured under two conditions (P1: Tera Harz Cure with a nitrogen generator (THC2) curing machine (Graphy, Seoul, Korea) and post-	MTT assay (MC3T3E-1 mouse pre-osteoblasts); cell viability measured spectrophotometrically (595 nm) after 7 and 14 days; viability (%) calculated vs. control (C+ = 100%) with cytotoxicity grading (none/slight/moderate/severe)	P1 (Tera Harz Cure with a nitrogen generator) showed high cytocompatibility at both time points, with cell viability comparable to the control and no significant differences, confirming its biocompatibility. In contrast, P2 (FormCure) exhibited moderate cytotoxicity, with significantly lower cell viability than P1 and the positive control at both 7 and 14 days, and a further reduction in cell survival over time, indicating mild cytotoxic effects.

			polymerised for 14 min; P2: 30 min on each side of the aligner (totaling 60 min), using the Form Cure (FormLabs Inc, Somerville, USA), ultrasonically cleaned, cut to 2×2 mm specimens, sterilised (121 °C).		
Iodice et al. (2024) [31]	Directly 3D-printed orthodontic aligner samples made from Tera Harz TC-85 DAC resin with different post-curing times (14, 24, and 50 min); Zendura FLX aligner material used as control.	In vitro	Aligner samples were incubated in centrifuged human saliva at 37°C for 14 days, and the collected extracts were tested for effects on cell viability. Additionall, fibroblasts were directly cultured on aligner samples and glass controls, and cell viability was evaluated after incubation.	Cytotoxicity was evaluated by measuring the metabolic activity of human gingival fibroblasts using the MTT assay. Cells were seeded in 96-well plates and exposed to aligner extracts. After incubation, MTT solution was added and metabolically active cells reduced it to formazan crystals, which were dissolved and quantified spectrophotometrically. Cell viability was expressed relative to the control.	All printed aligners, including the aligner control (Zendura FLX), showed significant cytotoxicity compared with the glass control, reflected by reduced human fibroblast growth ($p < .001$). Only the 50-minute curing time significantly decreased cell growth compared with the aligner control, while the 14- and 24-minute groups showed no significant differences. A significant negative linear trend indicated greater cytotoxicity with longer curing times.<br>In the MTT assay, 3D-printed aligners incubated with centrifuged saliva significantly reduced fibroblast viability, and pure saliva alone also decreased viability. Aligners immersed in DMEM showed a slightly lower reduction in viability, although this difference was not statistically significant.
Kim et al. (2024) [26]	3D-printed clear aligners made of photo-polymerizable polyurethane resin (Tera Harz TC-85, Graphy Inc., Seoul, Korea)	In vitro	3D-printed clear aligners were subjected to two cleaning methods to remove uncured resin: IPA (negative control) — immersion in 99.5% isopropyl alcohol, 1 min ultrasonic rinse, 5 min air-dry; and centrifuge — centrifugation with external heating at 23 ± 2 °C (RT) and 55 ± 2 °C (HT) at	L929 cells (ATCC CCL-1) cultured in RPMI 1640 with 10% FBS and 1% antibiotic–antimycotic were assessed for viability using the MTT assay. Cell viability was expressed as percentage of optical density relative to the blank.	All tested aligners showed high cell viability (>80%), with RT-6 and HT-4 groups exhibiting significantly higher values ($P < 0.05$). Centrifugal cleaning effectively removed uncured resin, and 55 °C for 2 min provided optimal resin removal while maintaining aligner translucency and cell viability.

			27.95g (500 rpm) for 2, 4, or 6 min. Specimens without treatment were designated NT. After cleaning, all aligners were cut, sterilized with EO gas, and extracts immersed in RPMI 1640 medium (0.2 g/mL) for 24 h at 37 °C for cytotoxicity testing.		
Bleilöb et al. (2025) [11]	3D-printed specimens fabricated from Tera Harz TA-28 aligner resin (Graphy Inc., Seoul, South Korea)	In vitro	Digitally designed circular 3D-printed resin specimens (Ø 10 mm) were fabricated in five thickness groups: 0.5 mm, 1 mm, 2 mm, 4 mm, and 6 mm. After printing, specimens were centrifugally cleaned, post-cured for 20, 30, or 60 minutes in a 95% nitrogen atmosphere, and washed in boiling water. Each thickness group (n = 6) was incubated in culture medium or saliva for 12 days with intermittent shaking to simulate clinical wear and obtain conditioned extracts. The extracts were then transferred to plates containing adhered human gingival fibroblasts for indirect in vitro cytotoxicity assessment.	Human gingival fibroblasts (HGFs; CLS Cell Lines Service GmbH) cultured in DMEM and exposed to conditioned medium/saliva from aligners of different thicknesses (n = 6 per thickness).<br>Cell viability was assessed after 48–72 h using the AlamarBlue assay (resazurin reduction to resorufin; fluorescence Ex 537 nm/Em 600 nm), normalized to negative controls (=100%). A reduction in viability >30% was considered cytotoxic according to ISO 10993-5; morphological evaluation was additionally performed (≥20% rounded or loosely attached cells indicating slight cytotoxicity).	A thickness-dependent decrease in HGF viability was observed after exposure to Tera Harz TA-28 specimens post-cured for 20 minutes; however, the reduction did not exceed 30%. Prolonged post-curing (30–60 min) did not improve outcomes, with the greatest decrease at 30 min and partial recovery at 60 min. Cells proliferated under all material-related conditions without morphological alterations.<br>Incubation in saliva significantly reduced proliferation: both unconditioned saliva (saliva not previously exposed to the material) and conditioned saliva (saliva pre-incubated with specimens for 12 days, potentially containing released substances) decreased cell growth, indicating that saliva itself exerted an inhibitory effect, which could be further influenced by material-derived components.

Bor et al. (2025) [28]	Samples of 3D-printed aligners produced from two commercial resins: Tera Harz TC-85 DAC (Graphy) and Clear-A (Senertek)	In vitro	Rectangular 3D-printed aligner specimens (10 × 12 mm; 0.6 mm thickness) were fabricated using an Ackuretta SOL printer (Ackuretta) (100 µm layer thickness, 45° angulation). Three experimental groups were established: (1) Tera Harz TC-85 DAC (Graphy) processed according to the manufacturer's instructions, including nitrogen-based post-curing (25 min; Tera Harz Cure THC2); (2) Clear-A (Senertek) post-cured in glycerin for 10 min (Curie Cure, Ackuretta); and (3) Clear-A with the same post-curing protocol as Group 1. All specimens were cleaned, thermally treated (boiling water, 100 °C, 1 min), disinfected in 70% ethanol, and incubated in DMEM (6 cm^2/mL) at 37 °C for 72 h to obtain extracts.	Cytotoxicity was evaluated using the human gingival fibroblast cell line (HGF; PCS-201-018™). Resin extracts were placed at different concentrations (100%, 50%, 25%, 12.5%, 6.25%). Cell viability was assessed at 24, 48, and 72 h using the XTT assay (Biological Industries), and results were expressed as percentage relative to untreated controls (100%). Additionally, real-time cell analysis (RTCA) was performed using the xCELLigence RTCA DP system (Agilent Technologies). Cells (1×10^4 cells/well) were monitored for 24 h prior to extract exposure, followed by 72 h continuous impedance-based measurement after addition of undiluted extracts (triplicates).	The XTT assay demonstrated that none of the tested resin extracts induced cytotoxicity, as cell viability remained above the 70% threshold defined by ISO 10993-5 at all time points. At 24 h, undiluted (100%) extracts resulted in approximately 75–80% viability across all groups, while diluted extracts (>50%) showed viability above 90%. At 48 and 72 h, no relevant differences in cell viability were observed among groups or concentrations. IC_{50} values could not be determined, as none of the extracts reduced viability by 50% within the tested range (6.25–100%), indicating IC_{50} >100%.<br>Real-time analysis using xCELLigence RTCA revealed a slight transient decrease in cell index within the first 24 h after extract addition, followed by recovery and continued proliferation. Doubling times were comparable among all groups, with no statistically significant differences, confirming the absence of relevant cytotoxic effects.

Table S2. Scoping Reviews (PRISMA-ScR) Checklist.

Preferred Reporting Items for Systematic reviews and Meta-Analyses extension for Scoping Reviews (PRISMA-ScR) Checklist

SECTION	ITEM	PRISMA-ScR CHECKLIST ITEM	REPORTED ON PAGE #
**TITLE**			
Title	1	Potential Cytotoxicity of Orthodontic Aligners in the Oral<br><br>Environment: A Scoping Review.	1-2
**ABSTRACT**			
Structured summary	2	Provide a structured summary that includes (as applicable): background, objectives, eligibility criteria, sources of evidence, charting methods, results, and conclusions that relate to the review questions and objectives.	1
**INTRODUCTION**			
Rationale	3	Describe the rationale for the review in the context of what is already known. Explain why the review questions/objectives lend themselves to a scoping review approach.	1-2
Objectives	4	Provide an explicit statement of the questions and objectives being addressed with reference to their key elements (e.g., population or participants, concepts, and context) or other relevant key elements used to conceptualize the review questions and/or objectives.	2
**METHODS**			
Protocol and registration	5	Indicate whether a review protocol exists; state if and where it can be accessed (e.g., a Web address); and if available, provide registration information, including the registration number.	2
Eligibility criteria	6	Specify characteristics of the sources of evidence used as eligibility criteria (e.g., years considered, language, and publication status), and provide a rationale.	2

SECTION	ITEM	PRISMA-ScR CHECKLIST ITEM	REPORTED ON PAGE #
Information sources*	7	Describe all information sources in the search (e.g., databases with dates of coverage and contact with authors to identify additional sources), as well as the date the most recent search was executed.	2
Search	8	Present the full electronic search strategy for at least 1 database, including any limits used, such that it could be repeated.	2-4
Selection of sources of evidence†	9	State the process for selecting sources of evidence (i.e., screening and eligibility) included in the scoping review.	2
Data charting process‡	10	Describe the methods of charting data from the included sources of evidence (e.g., calibrated forms or forms that have been tested by the team before their use, and whether data charting was done independently or in duplicate) and any processes for obtaining and confirming data from investigators.	3
Data items	11	List and define all variables for which data were sought and any assumptions and simplifications made.	2-4
Critical appraisal of individual sources of evidence§	12	If done, provide a rationale for conducting a critical appraisal of included sources of evidence; describe the methods used and how this information was used in any data synthesis (if appropriate).	8
Synthesis of results	13	Describe the methods of handling and summarizing the data that were charted.	3
**RESULTS**			
Selection of sources of evidence	14	Give numbers of sources of evidence screened, assessed for eligibility, and included in the review, with reasons for exclusions at each stage, ideally using a flow diagram.	3
Characteristics of sources of evidence	15	For each source of evidence, present characteristics for which data were charted and provide the citations.	3

SECTION	ITEM	PRISMA-ScR CHECKLIST ITEM	REPORTED ON PAGE #
Critical appraisal within sources of evidence	16	If done, present data on critical appraisal of included sources of evidence (see item 12).	2
Results of individual sources of evidence	17	For each included source of evidence, present the relevant data that were charted that relate to the review questions and objectives.	2
Synthesis of results	18	Summarize and/or present the charting results as they relate to the review questions and objectives.	3-5
**DISCUSSION**			
Summary of evidence	19	Summarize the main results (including an overview of concepts, themes, and types of evidence available), link to the review questions and objectives, and consider the relevance to key groups.	3-5
Limitations	20	Discuss the limitations of the scoping review process.	5-9
Conclusions	21	Provide a general interpretation of the results with respect to the review questions and objectives, as well as potential implications and/or next steps.	5-9
**FUNDING**			
Funding	22	Describe sources of funding for the included sources of evidence, as well as sources of funding for the scoping review. Describe the role of the funders of the scoping review.	9

JBI = Joanna Briggs Institute; PRISMA-ScR = Preferred Reporting Items for Systematic reviews and Meta-Analyses extension for Scoping Reviews.
* Where *sources of evidence* (see second footnote) are compiled from, such as bibliographic databases, social media platforms, and Web sites.
† A more inclusive/heterogeneous term used to account for the different types of evidence or data sources (e.g., quantitative and/or qualitative research, expert opinion, and policy documents) that may be eligible in a scoping review as opposed to only studies. This is not to be confused with *information sources* (see first footnote).
‡ The frameworks by Arksey and O'Malley (6) and Levac and colleagues (7) and the JBI guidance (4, 5) refer to the process of data extraction in a scoping review as data charting.
§ The process of systematically examining research evidence to assess its validity, results, and relevance before using it to inform a decision. This term is used for items 12 and 19 instead of "risk of bias" (which is more applicable to systematic reviews of interventions) to include and acknowledge the various sources of evidence that may be used in a scoping review (e.g., quantitative and/or qualitative research, expert opinion, and policy document).

From: Tricco AC, Lillie E, Zarin W, O'Brien KK, Colquhoun H, Levac D, et al. PRISMA Extension for Scoping Reviews (PRISMAScR): Checklist and Explanation. Ann Intern Med. 2018;169:467–473. doi: 10.7326/M18-0850.
